# Cell Guidance on Nanogratings: A Computational Model of the Interplay between PC12 Growth Cones and Nanostructures

**DOI:** 10.1371/journal.pone.0070304

**Published:** 2013-08-06

**Authors:** Pier Nicola Sergi, Iolanda Morana Roccasalvo, Ilaria Tonazzini, Marco Cecchini, Silvestro Micera

**Affiliations:** 1 Neural Engineering Area, The BioRobotics Institute, Scuola Superiore Sant’Anna, Pisa, Italy; 2 National Enterprise for nanoScience and nanoTechnology (NEST), Istituto Nanoscienze-National Council of Research (CNR) and Scuola Normale Superiore, Pisa, Italy; 3 Translational Neural Engineering Laboratory, Center for Neuroprosthetics & Institute of Bioengineering, School of Engineering, École Polytechnique Fédérale de Lausanne, Lausanne, Switzerland; University of California, San Diego, United States of America

## Abstract

**Background:**

Recently, the effects of nanogratings have been investigated on PC12 with respect to cell polarity, neuronal differentiation, migration, maturation of focal adhesions and alignment of neurites.

**Methodology/Principal Findings:**

A synergistic procedure was used to study the mechanism of alignment of PC12 neurites with respect to the main direction of nanogratings. Finite Element simulations were used to qualitatively assess the distribution of stresses at the interface between non-spread growth cones and filopodia, and to study their dependence on filopodial length and orientation. After modelling all adhesions under non-spread growth cone and filopodial protrusions, the values of local stress maxima resulted from the length of filopodia. Since the stress was assumed to be the main triggering cause leading to the increase and stabilization of filopodia, the position of the local maxima was directly related to the orientation of neurites. An analytic closed form equation was then written to quantitatively assess the average ridge width needed to achieve a given neuritic alignment (R^2^ = 0.96), and the alignment course, when the ridge depth varied (R^2^ = 0.97). A computational framework was implemented within an improved free Java environment (CX3D) and in silico simulations were carried out to reproduce and predict biological experiments. No significant differences were found between biological experiments and in silico simulations (alignment, p = 0.3571; tortuosity, p = 0.2236) with a standard level of confidence (95%).

**Conclusions/Significance:**

A mechanism involved in filopodial sensing of nanogratings is proposed and modelled through a synergistic use of FE models, theoretical equations and in silico simulations. This approach shows the importance of the neuritic terminal geometry, and the key role of the distribution of the adhesion constraints for the cell/substrate coupling process. Finally, the effects of the geometry of nanogratings were explicitly considered in cell/surface interactions thanks to the analytic framework presented in this work.

## Introduction

The outgrowth of neurites is a complex and multiscale phenomenon leading in human beings and animals to extremely specialized and effective neural structures both in the central [1] and peripheral nervous systems. Its study is fundamental to investigate the development of the nervous system, and is important in medical applications involving the regeneration of peripheral nerves after injuries [2]. Moreover, in the last decades, technological applications have made this field particularly attractive due to its implications in health care, especially for advanced prostheses for amputees [3], [4].

Although several types of neural cells have been used in the past for biological experiments, cells of pheochromocytoma cell line 12 (PC12) [5] have been widely used as a model to investigate neuronal differentiation and neuritic outgrowth. Indeed, they can reversibly adopt several neuronal characteristics upon exposure to nerve growth factor (NGF), resulting in finishing mitosis and extending protrusions, which are morphologically analogous to those of primary sympathetic neurons [6]. Biological experiments on flat substrates have shown different kinds of terminals, and “varicones” have been described when varicosities [7], [8] have been found together with growth cones (classic or non-spread), both in neuronal and PC12 cells [9], [10]. Biological experiments addressing aspects of the coupling between local extracellular topography and PC12 have also been performed using nanogratings (alternating submicron lines of ridges and grooves), which have influenced neural polarity [11], [12], cell differentiation [13], migration [14] and the modulation of focal adhesion maturation [15], [16]. Their ability to align neurites is promising for advanced biomedical applications such as the development of novel and more effective implantable neural interfaces and for improving existing solutions for peripheral nerve regeneration [17]–[22].

For this reason, over the last two decades, sophisticated tools have been developed in the field of computational neurosciences to simulate the physiology of neurons. These tools have reproduced experimental results in simple situations and have made it possible to plan new biological experiments.

Among others, CX3D (Institute of Neuroinformatics of ETH, Zurich) [23] is an open-source software written in Java used to model the growing of realistic neural networks in a three-dimensional physical space. Within this software, spheres and cylinders have mechanical properties and schematize cellular somata and neurites, allowing complex neural morphologies to be built. In addition, complex boundary conditions, as interactions among neighbouring objects and intra/extracellular diffusion, can be considered. CX3D is currently able to simulate a range of biological processes such as cellular migration and division, extension of axonal and dendritic arbours, interaction with extracellular cues, and formation of synapses. However, it currently does not allow biological contact-guidance experiments to be simulated [11], [15], [24].

The aim of this paper, therefore, was to develop a novel framework to model the outgrowth of PC12 neurites on nanogratings using an enhanced version of the CX3D source code.

The whole framework was validated by comparison with biological experiments [11], and further contact-guidance in silico simulations were performed to show how this tool could optimize both the design and planning of biological experiments on nanogratings. Moreover, the results show how simulation tools combined with theoretical models could also improve the design of regenerative electrodes [17].

**Figure 1:**
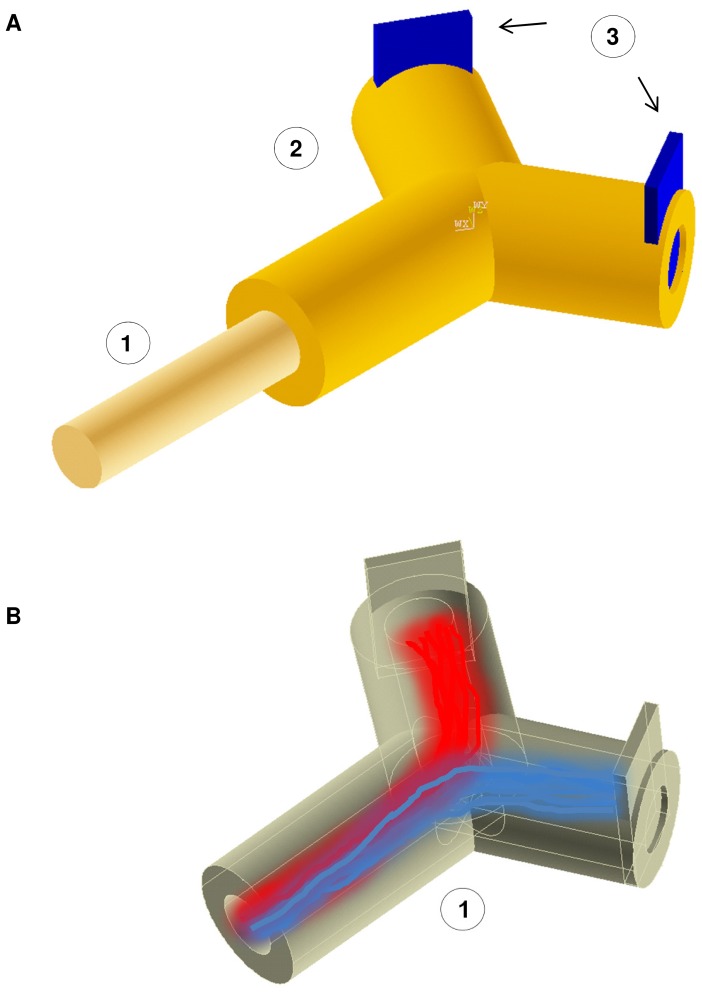
Regenerative interface (External and internal views). (A) Scheme of reciprocal positions of (1) Healthy stump of nerve; (2) Regenerative scaffold; (3) Contacts with active sites. The healthy stump of nerve is connected with the regenerative scaffold to allow the injured axons to regenerate and contact the active sites. From these contacts, electrical signals, closely related to the patient’s will of movement, can be achieved to drive neural prostheses. (B) Internal view of regenerative scaffold with active topographic constraints (e.g. nanogratings). This kind of structure could be able to split the beam of axons improving the selectivity of contacts with the active sites. Two different populations of axons (e.g. sensory and motor) are shown in red and blue. In this concept, the beam of axon was split by the synergy of nanotopography and chemical cues.

This particular kind of electrodes could be designed to selectively interface a high number of axons regenerating through holes, where active sites record action potentials and selectively stimulate groups of axons [17]. To enhance the selectivity of these electrodes the nervous fibres could regenerate within an active scaffold, where a synergistic action of nanotopography (e.g. nanogratings) and chemical cues can split the axon beam, allowing the separation of two families of fibres (e.g. motor and sensory fibres, see Figure 1).

## Materials and Methods

**Figure 2:**
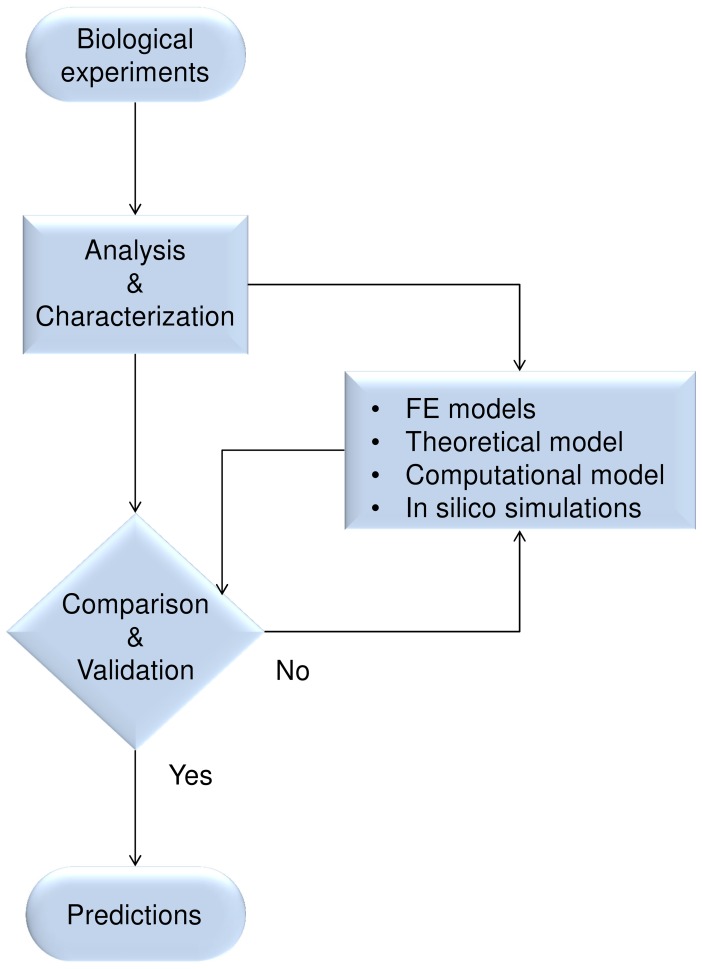
Logic flow of activities. Scheme of the activities carried out in this study: images of outgrowing neurites were taken from biological experiments performed on PC12, and analysed to investigate the morphology of terminals. Simple FE models were built from these morphological data to study, accounting for geometry and constraints, the course of stress at the intersection between collapsed growth cones and filopodia. Then, an analytic model was written to account for the nanograting geometry and to implement in silico simulations. In silico results were compared with biological data to validate the whole procedure and to provide predictions on more complex geometries.

The logic flow of this study is shown in Figure 2. First, optical microscopic images were extracted from biological experiments involving PC12 growing on nanogratings. These images were analysed to characterize the real geometry of neuritic terminals. Starting from these biological data, simplified finite element (FE) models were built to reproduce the main biological features of terminals (e.g., growth cones with non-spread collapsed appearance, adhesions, etc.). In addition, FE models were used to develop an analytic equation and to implement computational simulations within CX3D (referred to further on as “in silico simulations”). Finally, the results of biological experiments were compared with those of in silico simulations to validate the whole approach and to provide predictions in more complex cases.

### Cell Culture Imaging and Classification of Terminals

PC12 cells (CRL-17210, ATCC) were cultured and maintained at 37°C temperature and 5% CO_2_ in a RMPI growth medium supplemented with 10% Horse Serum, 5% Fetal Bovine Serum, 2 mM glutamine, 10 U/ml penicillin, and 10 µM/ml streptomycin. Cells were grown on three different cyclic olefin copolymer (COC) nanogratings: ridge depth and groove width were 250 nm and 500 nm, while ridge widths were 500 nm (period 1), 1000 nm (period 1.5) and 1500 nm (period 2) respectively. Images were also obtained from cell cultures on flat surfaces. Briefly, cells were differentiated by treatment with NGF at a final concentration of 100 ng/ml on different substrates, as previously reported [15]. Differential interference contrast (DIC) images were acquired with an inverted Nikon-Ti PSF wide-field microscope (Nikon, Japan) (oil immersion 40× 1.3 NA objective - PlanFluor, Nikon) after three or four days of culture. The bright-field optical microscopy images were loaded into ImageJ (within the plug-in NeuronJ, National Institute of Health, USA). Each experiment was repeated three times independently.

In accordance with [14], only cell protrusions emerging from cell bodies and longer than 10 µm were defined as neurites and analysed. Furthermore, neuritic terminals were classified with respect to the morphology of their growth cones (spread or non-spread) [9].

Neuritic trajectories were traced and their tortuosity (defined as the ratio between the actual neurite length and its straight length from the starting point to the ending point) was measured within MatLab ® (MathWorks, Inc, USA), and reported (mean ± standard deviation) for 117 neurites growing on nanogratings with period 1 µm, at 72 h, and for n = 3 different biological experiments.

### Immunocytochemistry: Tubulin and Actin Staining

Control experiments were run to investigate and confirm the different morphologies of PC12 neuritic terminals [8] on nanogratings, by looking at their cytoskeletal composition and morphology. PC12 cells were cultured and differentiated up to 4 days with NGF (100 ng/ml) on nanogratings (ridge width 500,1000,1500 nm), as previously reported in [15].

Cells were fixed in 4% paraformaldehyde and then immuno-stained with anti-β Tubulin III antibody (Sigma, T2200; 6 µg/ml) and phalloidin-Alexa Fluor 647 (Invitrogen), in GDB buffer (0.2% gelatin, 0.8M NaCl, 0.5% Triton X-100, 30mM phosphate buffer, pH 7.4) [25], [26] .

Samples were then washed, incubated with Alexa Fluor 488-secondary antibody (Invitrogen) and mounted with Vectashield medium (Vector laboratories, Burlingame CA, USA).

Fluorescent samples were then examined at a TCS-SP laser scanning confocal microscope (Leica Microsystems, Germany) with a 40 x 1.4 NA objective (Plan Apochromat, Leica), and high resolution three-dimensional Z-stacks of PC12 cells were acquired. Images were then processed by ImageJ (National Institute of Health, USA).

### Finite Element Models

The majority of growth cones showed a non-spread collapsed appearance [9] on both flat and nanopatterned surfaces. Therefore, a non-spread geometry was used to implement FE models in this study.

**Figure 3:**
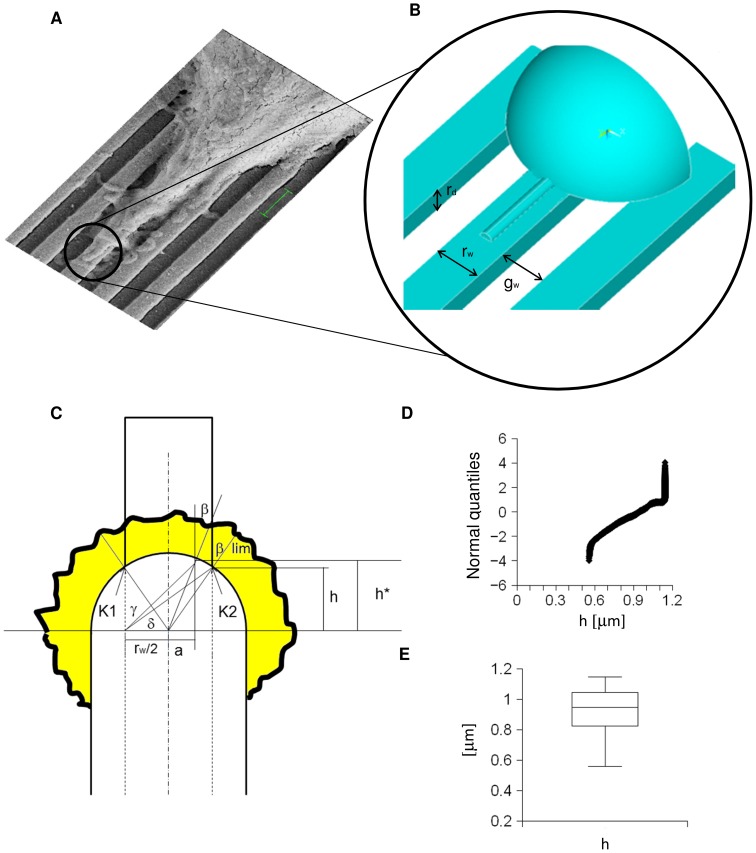
From biological experiments to computational models. (A) A SEM image of filopodia emerging from a non-spread growth cone (bar = 1 µm). (B) FE model of a non-spread growth cone showing a simplified geometry together with an emerging filopodium. The set of parameters necessary to characterize the nanograting geometry is also shown: ridge width r_w_, groove width g_w_, and ridge depth r_d_. (C) Bidimensional model of interactions between non-spread growth cone and ridge surface. Point K2 (together with K1, symmetric with respect to the centreline of the filopodial shaft) shows the limit angle β_lim_. The quantity h^*^ was connected to the actual intersection angle through a fraction of the ridge width (a). (D,E) Quantile-quantile plot of the quantity h as derived from in silico simulations, together with its box plot.

Since tensile stress promotes the initiation of new neurites [27], stress was assumed to mainly drive the neuritic outgrowth. As a consequence, this quantity was studied to predict both direction and alignment of neurites on nanogratings, which were described through simple geometrical features such as ridge depth (r_d_), groove width (g_w_) and ridge width (r_w_) (see Figures 3A–B).

In general, axons show a complex response to mechanical stimuli: first, a fast elastic response, then a slower passive viscoelastic behaviour [28], [29], and finally an active behaviour due to molecular motors [30]. In this work, since the protrusion-retraction cycles were faster than the outgrowth velocity of the main neuritic shaft [31], the response of filopodia was assumed to be mainly elastic. As a consequence, filopodia were characterized by two parameters (Young Modulus E = 106 Pa, and Poisson ratio ν = 0.47 [32]), and viscous effects were neglected.

In Figures 3A,B, a simple configuration, accounting for a non-spread collapsed growth cone [8], [9] with an emerging filopodium, was shown in comparison with the real geometry of a scansion electron microscopy (SEM) image. The growth cone was approximated with a quarter of sphere, while the filopodium was stylized with a half of a circular cylinder. Moreover, the growth cone was assumed to widely adhere [33], [34] to the substrate, while the filopodium was assumed to be fixed only through tip adhesions [35].

Furthermore, traction stresses, due to the reconfiguration of the neuritic cytoskeleton [36], were modelled through an imposed shortening at the contact area between the non-spread growth cone and neurite. As a consequence, the ending part of the filopodium (ending line) was totally constrained to the substrate (i.e., all degrees of freedom were set to zero), while the filopodial main shaft and the bottom surface of the growth cone were left free to shift only along their longitudinal axis.

Since any propagation of elastic waves was neglected, a quasi-static analysis of a nearly incompressible material was carried out using a three-dimensional 4-node tetrahedral structural solid (ANSYS ® Academic; Ansys, Inc. Canonsburg, Pennsylvania, USA). The mesh of volumes was achieved by using four node elements, which accounted for three translational degrees of freedom, together with the volume change rate.

To resume the local state of stress of each element, the Von Mises (VM) measure was chosen, because, within this metric, normal and shearing stresses were both considered. Indeed, VM stress was defined as:
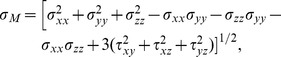
 where 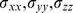 were normal stresses (respectively in x,y,z direction), while 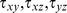 were shearing stresses.

The previous simple configuration was used to investigate whether the maximum VM stress depended on the length and orientation of the filopodium.

In addition, a more complex configuration was considered to study filopodia emerging with different orientations: the growth cone was approximated with a quarter of sphere, while filopodia were stylized with three half circular cylinders having a length of 0.1, 0.5, 1 times the growth cone radius. This analysis was performed to compare the influence on the stress field of both filopodial length and orientation, when three different filopodia shared the same contraction of the main neuritic shaft. This case showed a strong similarity with the previous configuration accounting for a single filopodium.

In conclusion, FE simulations qualitatively provided the location of the VM stress field varying the length, number and orientation of filopodia: in both cases maximum stresses were located on the shorter and stable filopodia. As a consequence, a growth cone with non-spread collapsed appearance and one emerging filopodium was studied to obtain a bidimensional analytic model.

### From the Filopodium to the Neuritic Path

The outgrowing neurites were iteratively described as a sequence of oriented straight segments. The first tract was assumed to be an enlargement of the main filopodium, directly emerging from the collapsed growth cone and following a Laplacian distribution over the interval [11]. To account for the influence of nanotopography on the other tracts, a system of difference equations (1) was used [37] :
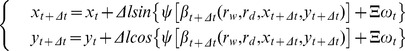
(1)where 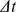 was the discrete interval of time in which the neurite extended of 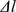, 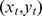 was the geometric position of the neurite tip (corresponding to the growth cone) at time t, ω_t_ was a random variable with a Gaussian distribution, and 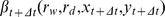 was the term accounting for the angular influence of the surface geometry with respect to the actual position 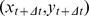 of the growth cone at time 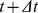. Finally, r_w_ and r_d_ were the ridge width and depth, Ξ = 75, and the explicit form of the 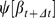 function was expressed in Eq. (2):
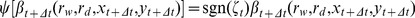
(2)where, the function 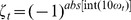 accounted for the symmetric possibility of the tip to turn on the right or on the left.

In Figure 3C, starting from the inside, two main half circles represent the border of the neuritic tip and the external shape of lamellopodia. The projections of ridges and grooves are also shown as alternated and juxtaposed rectangular areas. A symmetric ridge, having its mean line passing through the centre of the neurite, was chosen as example. Although different geometrical combinations between collapsed growth cones and ridges were possible (e.g., the contact between the non-spread growth cone and two or more ridges), more complex cases were led back to this one, because the main ridge, acting as a filter, mainly influenced the global orientation of the outgrowing neurite [31]. In the case of many principal ridges, each ridge was assumed to independently interact with the emerging filopodia, which were modelled as straight segments radiating from the centre of the growth cone.

The VM stress was assumed to promote filopodial extension, thus the most probable simulated filopodia had local VM stress maxima at the intersection with the non-spread growth cone. As a consequence, these limit filopodia passed through points K1 and K2, which were on the ridge border (see Figure 3C), and had a null length on the ridge.

Real emerging filopodia, instead, as well as having local maximum values of VM stress, laid on the surface of the main ridge. This condition further constrained their alignment to angles 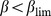, requiring that 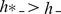. To assess quantity 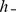, the mean equivalent radius of 7 non-spread growth cones was measured from optical microscopy images. They were loaded within NeuronJ plug-in to trace neuritic ends and analysed using a bidimensional CAD program. Finally, the mean equivalent radius resulted in 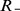 =  1.14 µm. In particular, 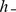 represented the y-coordinate of intersection between the mean collapsed growth cone (with radius 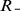) and the planar projection of the ridge width. This quantity was computationally approximated (using a stylized non-spread circular growth cone with radius 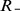) within CX3D (see following paragraph). In Figures 3D–E, both the distribution and the median value (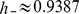) of h were shown. The simplest condition for having 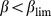 was chosen, so 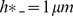 , and the mean value of the angle 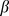 was expressed as:
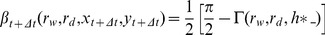
(3)where the function 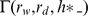 accounted for the ridge width and depth (in micrometers). A simple form of this function was written in Eq.(4) to decouple the influence of ridge width and depth:
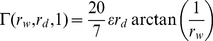
(4)where ε was a numerical parameter, achieved through fitting of biological data [15], while 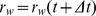 and 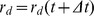 were geometrical parameters perceived by the growth cone in the current position.

### Implementation of CX3D Simulations

The source code of CX3D (http://www.ini.uzh.ch/projects/cx3d/) was enriched and modified to reproduce and design biological experiments on nanogratings. The standard code was improved through the addition of classes and methods to define an internal pattern (grating) accounting for biological contact-guidance experiments through Eqs. (1–4). The process of axonal guidance was assumed to be strongly affected by the growth cone dimensions, but CX3D currently lacks the classes to model the growth cone as a real physical element. To overcome this drawback, a circumference (with radius 1.14 µm, see previous paragraph) was used as a virtual growth cone to consider the interaction between growth cone and nanograting. The centre of this circumference was superimposed to the point mass of the distal segment of the neurite. As a consequence, the contacts between grating and growth cone were placed on the neurite tip and depended on their current reciprocal position. All possible interactions (on simulated gratings with period 1) were included in the following cases:

case 1: the point mass of the growth cone was placed within a groove and the growth cone overlied on two ridges. In this case, two angles (β_1_ and β_2_ ) quantified the influence of grating on the growth cone advancement. These angles were defined as the β angles of the segments passing near K1 and K2 (see Figure 3C). In this case the current β angle was randomly chosen between β_1_ and β_2_.

case 2: the point mass of the growth cone was placed within a ridge or on one of its borders. In this case, the current value of the angle β was unambiguously defined.

In both cases Eqs. (3,4) were used to calculate the current β angle, during advancement of the neurite.

A Java class was written to implement in silico simulations: the main method contained the instructions to define cell and grating geometry [11] within the CX3D physical space. In silico simulation started when two cell bodies, with a diameter of 10 µm and extending neurites, were placed on the virtual grating.

At the beginning of in silico simulation, neuritic processes extended from virtual somata to a length of 11.38 µm. According to [11] this initial length was assumed to be equal to that of neurites growing on flat surfaces. The neuritic point mass was then moved according to Eqs. (1,2), to account for the grating influence on the growing neuritic tip. The advancing speed was set equal to 20 µm/h, according to the biological data [38] for PC12 cells. When the neuritic length overcame this threshold, in silico neurites underwent extension and retraction cycles, at different speeds, until the end of CX3D simulation, according to [11].

A Java method was used to generate a length value from a Gaussian distribution with mean 18.98 µm and standard deviation of 2.65 µm. The mean of in silico distribution coincided with the mean value of biological lengths (at 12, 36, 60 hours), while the standard deviation was set equal to the smallest observed deviation. Then, for all steps, neurites cyclically extended or retracted to reach a length with a velocity derived from experimental data of cell cultures. The frequency of oscillations was implemented through a counter to reproduce the biological dynamics [11].

For each time point, biological experiments and in silico simulation had a similar range of variability. In silico neurites extended from somata into extracellular space until they reached a length that was nearly comparable with the biological values observed at the same time.

The reliability of the imposed dynamics was also studied through statistical comparison between mean experimental values of lengths (at 12, 36, 60 hours) and in silico values (at 6, 12, 18, 24, 30, 36, 42, 48, 54, 60 hours). For experimental lengths, a Shapiro-Wilk normality test resulted in W = 0.9912 and p = 0.821, while for the in silico ones in W = 0.8951 and p = 0.1933. As a consequence, both groups were assumed to have a Gaussian distribution, and a Welch t-test (two sided, independent samples) was used for statistical analysis and resulted in p = 0.3504.

In addition, the time courses of neuritic alignment and tortuosity were used to assess the reliability of in silico simulations with reference to biological experiments. To extract these parameters, a Java method was written to return output files containing values of virtual neurite alignment and tortuosity along time. The tortuosity was assessed using a standard procedure [37], while the alignment was calculated as the absolute value of the angle between the main direction of grating and the segment linking the starting and the ending points of the neurite.

## Results

### Morphological Characterization of PC12 Terminals

**Figure 4:**
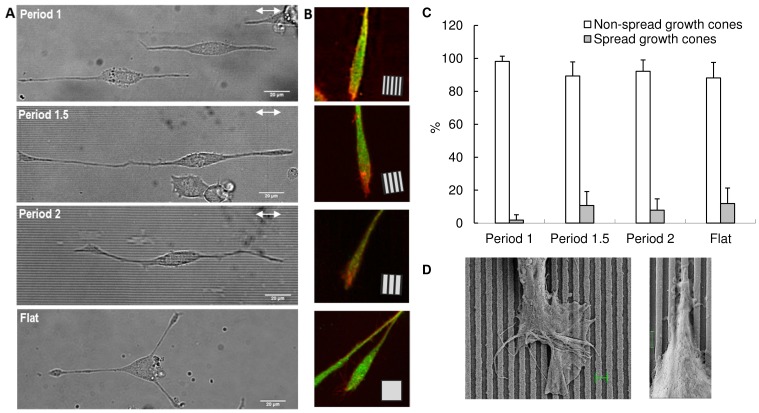
Analysis of neuritic terminals. (A) Typical bright field images of PC12 cells differentiated by NGF on period 1, 1.5 and 2 gratings and on flat substrate (from the top, respectively). White arrows: grating direction; bars = 20 µm. (B) Typical confocal images of the morphological aspect of terminals with non-spread growth cones: PC12 neurite terminals grown on period 1, 1.5 and 2 gratings and on flat substrate, and stained for β3-Tubulin (green) and actin (red). Each panel side = 25 µm; square inset: grating direction. (C) Analysis of PC12 neuritic terminals over different nanogratings (periods 1, 1.5, 2 µm) and flat substrate: terminals were characterized with respect to their morphology as spread or non-spread growth cones. The analysis was carried out on 323 terminals. (D) SEM images of PC12 growth cones on period 1 nanogratings: a spread growth cone (left), magnification = 3110 X, bar length =  1 µm; a non-spread growth cone, presenting lateral transient processes (right), magnification = 12550 X, bar length =  1 µm.

Optical microscopic images of PC12 growing on three different types of gratings were analysed and compared to those of cells growing on flat substrates (Figure 4A). After three/four days of culture, two types of terminals were observed at the end of the neuritic processes: a first type had varicosities, which were more or less evident and close to the neuritic tip, where a non-spread growth cone was present; a second type ended with a spread growth cone.

This optical analysis was supported, according to [8], by immunostaining experiments (Figure 4B), which highlighted both the morphology and cytoskeletal organization of neurite terminals on nanogratings and flat substrate. In particular, Figure 4C shows that, on flat surface, most of the observed neurites ended with non-spread growth cones (88%), while the others ended with spread growth cones (12%). Similarly, on nanogratings with period 1.5 µm, the percentage of non-spread and spread growth cones was 89% and 11% respectively, while on nanogratings with period 2 µm percentages were 92% and 8%. Finally, on period 1 µm, the percentage of non-spread growth cones increased up to 98% and spread growth cones decreased to 2%. Therefore, optical analysis supported the use of non-spread growth cones with a collapsed appearance to perform FE simulations, theoretical models and in silico simulations.

To clarify the morphological differences between the two types of terminal, two SEM images of spread (left) and non-spread (right) growth cones on period 1 nanogratings are shown in Figure 4D.

### Finite Element Models

Although the FE models were geometrically simple, they were able to qualitatively characterize the constrained contraction of the neuritic cytoskeleton. Indeed, the FE models aimed at reproducing biology, and each filopodium was fully constrained at its tip (tip adhesions) [35], while the collapsed growth cone was constrained to the substrate below (ridge surface) [33].

**Figure 5:**
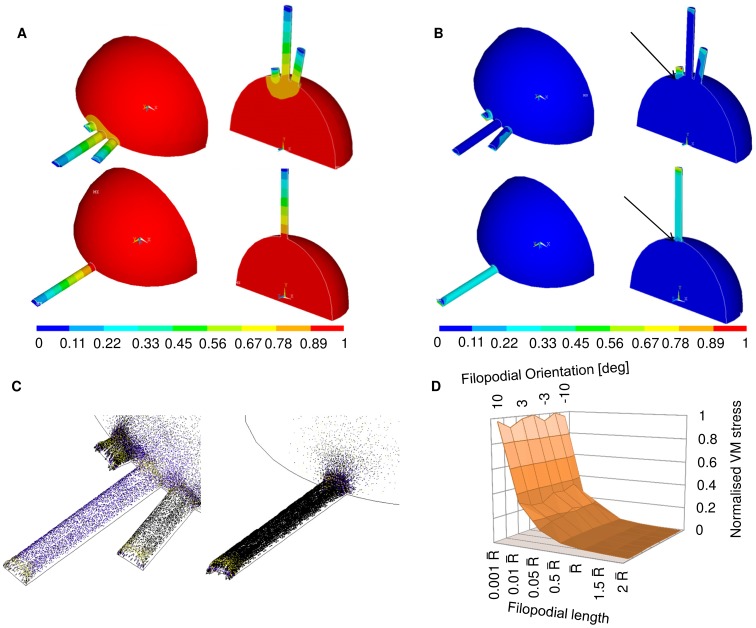
Finite Element models of non-spread growth cones. (A) Displacement field for FE models of a non-spread growth cone with three (up) and one (down) emerging filopodium. The displacements were normalized over the global maximum over the whole non-spread growth cone, which accounted for the contraction of the neuritic cytoskeleton. The distribution of displacement was linear and similar among different filopodia (up). It was also similar to the displacement distribution of the model with one emerging filopodium (down). (B) Von Mises stress field for FE models of a non-spread growth cone with three (up) and one (down) emerging filopodium. VM stresses were normalized over the maximum stress at the tip of the shortest filopodium. Unlike the displacement field, the VM stresses varied among filopodia of different lengths. In particular, the course of VM along the shortest filopodium was similar for both models with three and one emerging protrusion. Arrows pointed the investigated local maxima of VM stress at the intersection between non-spread growth cones and filopodia for both models (up and down).(C) Vector plots of principal stresses for both models; left: magnification of the vector field for the model with three emerging filopodia; right: magnification of the vector field for the model with one emerging filopodium. In this case also, both fields were similar but scaled. (D) Modular surfaces accounting for the variation of intersection VM stress with filopodial orientation and length. The plot accounted for angular variation in the range (−10°, 10°) and different length of filopodia in the range 0.001
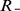
–2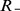 where 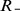 was the radius of the non-spread growth cone. All values were normalized on the interface VM stress at 0° for a length of 0.001
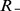
_._

In order to generalize the analysis, the field of displacement was studied for non-spread collapsed growth cones with three (Figure 5A up right, left) and one (Figure 5A down right, left) emerging filopodia. This analysis was used to highlight similarities between different filopodia (Figure 5A up right, left), and similarities between FE models with three and one emerging filopodia.

In addition, the VM stress field was investigated for both models. In Figure 5B the local maxima at the intersection between filopodia and non-spread growth cone are shown using black arrows.

The similarity between the two models is also shown through the principal stress field (Figure 5C). Indeed, the shortest filopodium of the FE growth cone with three filopodia (Figure 5C left) had a vector field, which was similar, even if scaled, to that of Figure 5C right. As a consequence, the FE model with an emerging filopodium was used to investigate the course of the local maximum VM stress, varying both orientation and length of filopodia.

In Figure 5D, the values of VM stress are shown for filopodia with different orientations (−10°, 10°) and increasing lengths ranging from 0.001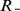 to 2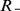. All values were normalized over the intersection stress at 0° for a length of 0.001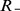.

The course of data was constant with respect to the axis of orientation, showing that the VM stress was independent from this parameter. On the contrary, the course of the modular surface was highly non-linear with respect to the length axis, showing the importance of the length of the filopodium to maximize the VM stress at the intersection between non-spread growth cone and filopodia.

In conclusion, the maxima VM stresses in both models were located at the shortest and most stable (e.g., on the main ridge) filopodia.

### The Analytic Model

The local maximum VM stress, at the intersection between non-spread growth cone and filopodium, was assumed to be the main triggering cause of the directional growth of neurites on nanogratings. FE simulations showed a clear dependence of this local maximum VM stress from filopodial length. Using a simple analytic model, the nanograting geometry (e.g., ridge width and depth) was also accounted for to specify the position of the most stable filopodium.

**Figure 6:**
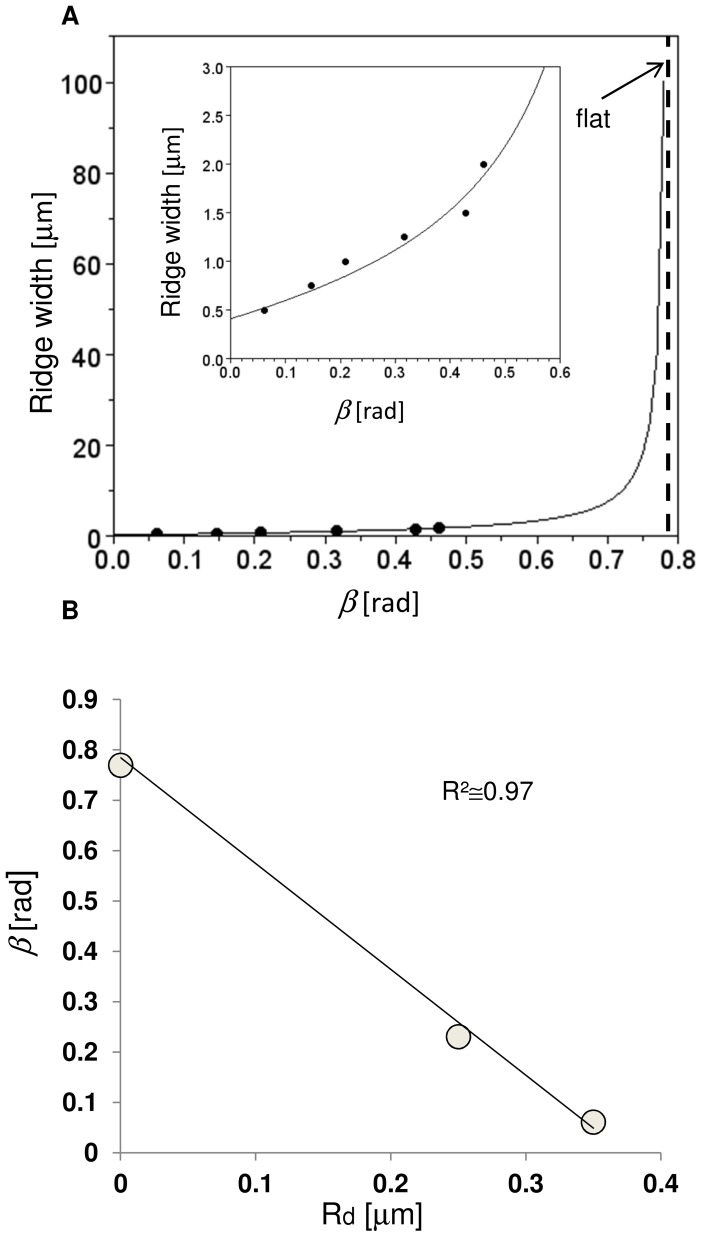
Analytic model. (A) Quantitative prediction of the ridge width to achieve a given mean alignment angle β. All experimental points were obtained keeping the ridge depth constant (350 nm) while the ridge width varied in the range 500–2000 nm. Inset: magnification for small values of β. (B) Influence of the ridge depth on the value of β when the ridge width was kept constant and the ridge depth varied between 0 (flat) and 350 nm.

In particular, using Eqs. (3,4), experimental data (mean alignment of neurites) were fitted [11], [15] (to quantify the parameter ε) and expressed as a function of both ridge width and depth. Furthermore, the performances of the analytic model were shown in Figure 6A to quantitatively predict the ridge width needed to achieve a given mean angle of neuritic alignment. In this plot, the previous analytic relation was inverted and the ridge width was fixed at 350 nm, to obtain the relation 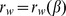 (bold line), that was able to fit (R^2^ ≅ 0.96) experimental mean values. The R^2^ value was the square of the correlation between the experimental data and the predicted values. This value was referred, at the same time, to all data points (mean values of experimental measurements). Then, Eq. (3) was able to model the average behaviour of 311 cells. In Figure 6A, the flat surface was considered as a limit case (vertical asymptote), when the ridge height decreased towards zero and the ridge width increased without bond. The inset showed a magnification of the analytic model performances for small values of the ridge width.

Figure 6B shows that 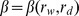 was able to predict the influence of the ridge depth while keeping the ridge width constant (500 nm). The β angle was defined as the angular difference between the main direction of nanograting and the actual direction of the outgrowing neurite [11]. Experimental points corresponded to different values of the ridge depths (0 nm (flat), 250 nm, 350 nm). Furthermore, Eqs. (3,4), once the ε parameter had been quantified using equation 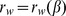, provided a negative trend near to the experimental data (R^2^ ≅ 0.97), when the ridge depth increased.

### Guided Neuritic Outgrowth: in silico Simulations within CX3D

**Figure 7:**
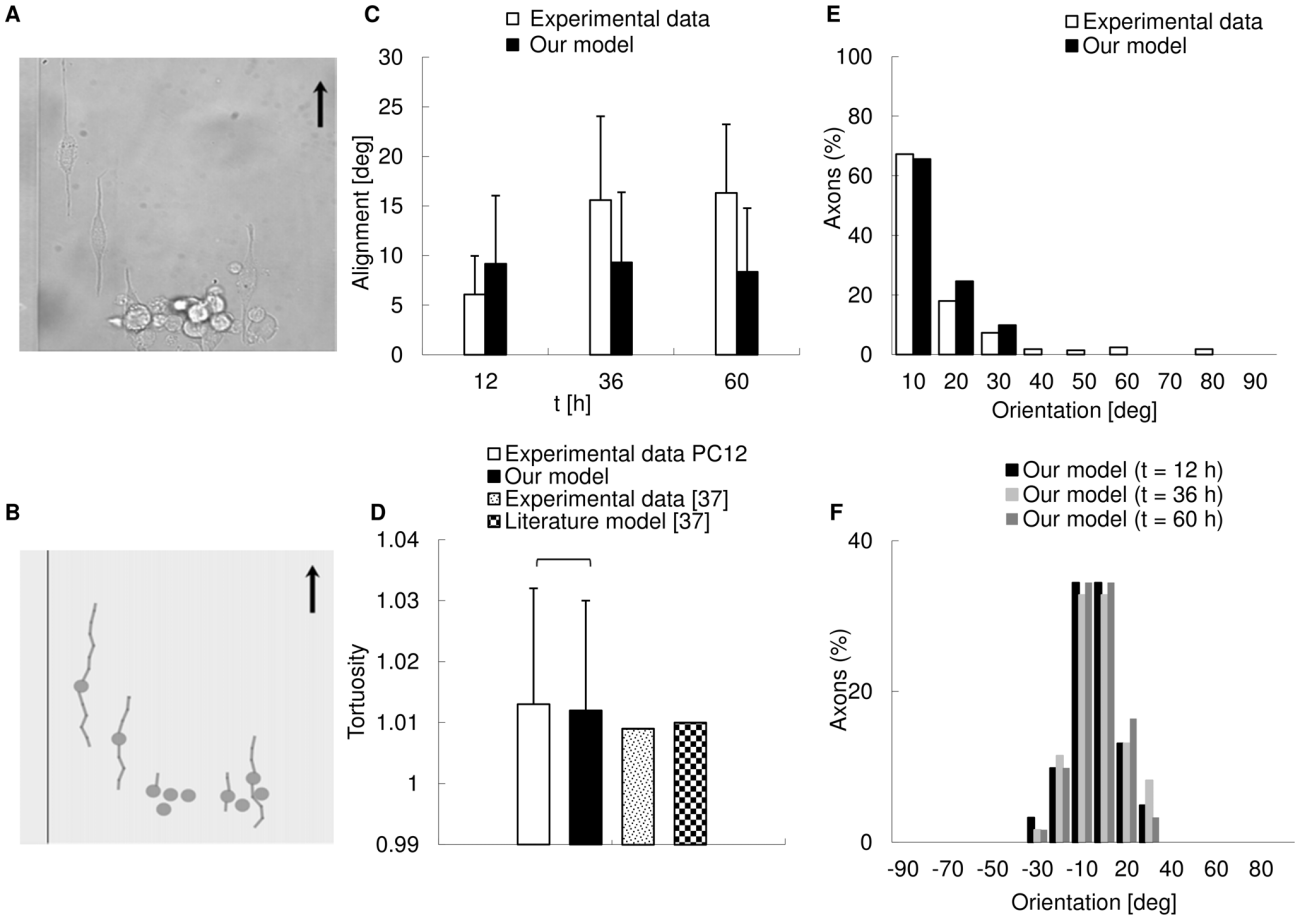
In silico predictions of biological experiments. (A) DIC image of a cell culture acquired with an inverted Nikon-Ti PSF wide field microscope: PC12 cells were differentiated on a nanograting with period 1 and the black arrow shows the main direction of the grating. (B) Biological experiment (see A) simulated in CX3D. In silico cells were placed on a virtual nanograting with the same geometry of biological experiments and neurites grew according to the framework described by Eqs. (1–4). The black arrow indicates the main direction of the grating. (C) Comparison between biological and in silico alignments. Biological data were collected at t = 12, 36, 60 h, while in silico values were kept at t = 6, 12, 18, 24, 30, 36, 42, 48, 54, 60 h (61 neurites, error bars = standard deviations). Then, the mean biological and in silico values were compared and a Welch t-test resulted in p =  0.3571 (no significant difference). (D) Comparison between experimental, theoretical [37] and in silico mean tortuosity (117 neurites). No significant difference was found in PC12 between experimental and in silico values of tortuosity (Wilcoxon rank sum test, p = 0.2236). (E) Control of neuritic orientation for neurites on a period 1 nanograting in biological experiments [12] and in silico simulations. The percentages of neurites aligned to the grating axis within different angular ranges were reported: in both cases, most of the neurites aligned within 20° with respect to the main grating direction. The range of orientations was similar in both cases. (F) Temporal evolution of orientation control for neurites in CX3D physical space. The orientation of neurites was reported at t = 12, 36 and 60 h and showed small differences over time. Most neurites aligned to the main grating direction within small angular ranges (±20°) for any sampled time.

In Figure 7A, a DIC image, acquired with an inverted Nikon-Ti PSF wide field microscope, is shown, where PC12 cells were differentiated over a nanograting (period 1). In Figure 7B, the same biological experiment was simulated within CX3D by placing a cell population on a virtual grating and maintaining experimental parameters (r_w_ = g_w_ = 500 nm, r_d_ = 250 nm). In both cases, black arrows show the main orientation of the anisotropic surfaces. In Figure 6C, the comparison between biological and in silico (n = 61, mean values ± standard deviation) alignments is shown.

At first, in silico alignment had a mean value greater than the biological one (t = 12 h), yet within the experimental error bar. Later, at t = 36 h, the mean in silico value was slightly lower than the experimental one, but again inside the range of variability. Finally, at t = 60 h, in silico alignment was lower than the biological one, and at the border of the range of variability.

Moreover, this last value was also lower than the previous ones, suggesting that in silico neurites quickly aligned with respect to grating direction over time.

A standard statistical approach was used to test whether in silico simulations were able to globally approximate biological experiments. Experimental alignments at times t = 12, 36, 60 hours, were compared with mean simulated alignments at t = 6, 12, 18, 24, 30, 36, 42, 48, 54, 60 hours. In particular, for experimental alignments, a Shapiro-Wilk normality test resulted in W = 0.8017 and p = 0.1186, while for in silico ones W = 0.9671 and p = 0.8632. Then, both groups were approximated by a normal distribution, and a Welch t-test (two sided, independent samples) resulted in p = 0.3571.

Figure 7D shows the results of experimental and in silico tortuosity obtained for PC12 cells, and [37] for primary cells. In particular, in silico neurites (n = 117) had a tortuosity of 1.012 ± 0.018, which was close to that experimentally observed (n  = 117) and equal to 1.013 ± 0.019 (data were expressed as average value ± standard deviation). No significant difference was found between experimental and in silico values (Wilcoxon rank sum test, p = 0.2236).

Similarly, for primary nerve cells [37] simulations and experiments resulted in mean tortuosities of 1.01 and 1.009, respectively.

The abilty to control the neuritic orientation was shown, for a nanograting with period 1, in Figure 7E, where experimental [12] and in silico results were compared. According to biological experiments, the nanostructure led 67% of neurites to align to the main grating direction within 10° and over 90% within 30°. The other protrusions (∼ 7%) were aligned within a 30°–80° range. Similarly, when the same analysis was carried out for in silico neurites growing on a period 1 virtual nanograting, most of the neurites (65%) were aligned within 10°, and over 90% within 20°, while the other cell processes (almost 10%) were oriented in a 20°–30° range with respect to the main direction of grating.

The time evolution of the orientation control is shown in Figure 7F: virtual neurites grew on a period 1 nanograting within the CX3D physical space, and the distribution of the neuritic orientations was plotted at t = 12, 36, 60 h.

First, at t = 12 h, most neurites were aligned to the grating axis within ±10° (∼ 69%) and over 90% within ±20°. All the remaining protrusions were oriented within ±30°.

Then, at t = 36 h and t = 60 h, the percentages of neurites for each angle bin slightly varied. This suggested that in silico neurites had a poor variability over time in terms of angular orientation. Moreover, for any sampled time, most neurites aligned to the grating axis within small angular ranges (almost 70% within ±10° and over 90% within ±20°), according to biological experiments [12].

These results supported the use of Eqs. (3,4) to perform in silico simulations of complex interactions between nanopatterned surfaces and neuritic processes.

**Figure 8:**
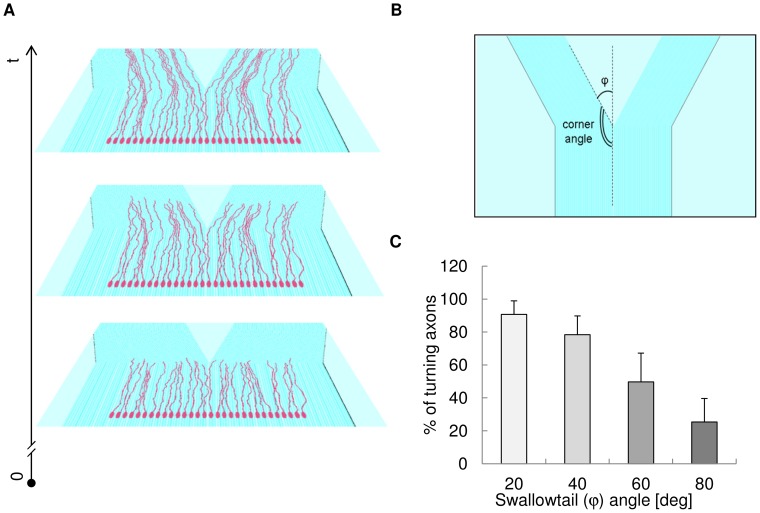
In silico simulations of beam splitting experiments. (A) In silico simulation of a group of 30 cells on a swallowtail grating. Different phases of neuritic outgrowth are shown on the planes (in perspective) over time (t). In simulations, geometric and fasciculation effects were considered together. (B) Geometrical angles to model the swallowtail: the φ angle accounted for the steepness of the change between the straight grating and the following bifurcation. (C) Percentage of turning axons with different values of the φ (swallowtail) angle. This percentage decreased as the φ angle increased, in agreement with literature [39].

In Figure 8A, a contact-guidance experiment on a grating is simulated within CX3D to obtain a specific neuritic pattern (neural beam splitting). In silico cells (n = 30) were “cultivated” on a swallowtail grating, and the neurites, extending from each soma, were guided by the substrate topography along the main direction of local anisotropy. The final path of each neurite was then influenced by the actual position on the nanograting when the substrate branched into swallowtail, and neurites were able to turn on the right or on the left. Moreover, fasciculation effects were considered in CX3D standard code and cellular interactions were implemented through repulsive and attractive components: the first class prevented the overlapping of cells, the second accounted for the effect of adhesion molecules.

As a consequence, neurites interacted with each other and modified their direction of advancement not only by following the grating influence, but also by avoiding overlaps with the near cellular protrusions and forming fascicular structures, roughly resembling axonal fascicules.

In addition, four different swallowtail geometries were implemented (with tail angles φ of 20°, 40°, 60° and 80°) to assess the influence of topography on the turning ability of the axons (Figure 8B).

On the basis of experimental results on different substrates (e.g. flat and corridors) [39]–[41], the turning ability was determined by the angle of approach between the neuritic tip and the ridge border, thus the axons that failed to turn stopped in proximity of the corner.

In silico results were shown in Figure 8C: the axons were able to turn on all tested geometries, but the percentages of turning axons were ∼ 91%, ∼ 78%, ∼ 50% and ∼ 25% for tail angles of 20°, 40°, 60° and 80° respectively. These results showed that the turning frequency decreased as the tail angle increased (and the corner angle decreased), accordingly to the experimental results reported in literature [39].

## Discussion

PC12 neurites growing on nanogratings and flat substrates were investigated through optical analysis of DIC and SEM images, further strengthened by immunocytochemistry. As a result, most of the growth cones showed a non-spread collapsed appearance (Figure 4B).

Nevertheless, non-spread growth cones were regularly involved in pathfinding through a refined sensing of substrates, consistently with biological experiments on nanopatterned [13], [15], [16] and flat substrates [8], [9].

In particular, since nanogratings induce a high degree of anisotropy, the scanning mechanism of the filopodia highly depended on their current position with respect to the principal direction of nanopatterns, and the filopodia were grouped together in two different subsets: non-aligned and aligned [31]. Indeed, only filopodia entirely extending over the ridge surfaces had a continuum contact zone and enlarged through a robust F-actin network [31].

Although this simple mechanism seemed to explain the reason why the neurites tended to avoid unaligned tracks, unfortunately it was not able to quantify the mean neuritic misalignment [11], nor its changes related to the dimensions of nanograting [15]. As a consequence, this work aimed at quantifying both these issues starting from the reciprocal position between non-spread growth cones and ridge surface.

The local maximum VM stress, at the interface between filopodia and non-spread growth cone, was assumed to be the main cause triggering the biophysical reactions leading to the outgrowth of neurites. This mechanism had the easiness of a “search and capture” mechanism [31], but a much greater ability to orient the path of the neurites. Accordingly, the size of non-spread growth cones, the dimensions of ridges, together with the characteristics of growth cone/substrate coupling, emerged as the main factors involved in neuritic pathfinding.

Furthermore, advanced issues, such as the balance between deterministic and stochastic effects [42], also emerged as being fundamental to the building of the neuritic paths.

This framework was able to implement direct and inverse in silico simulations with a chosen number of neurites and with single or combined substrates of any complexity. Direct simulations, as previously shown in the results section, were calibrated through experimental data to approximately reproduce the biological behaviour of PC12 neurites.

However, it was difficult to clearly define what “to reproduce biological experiments” generally means: “optical” likeness on its own (as shown in Figures 7A–B) was necessary but not sufficient to provide suitable in silico simulations.

As a consequence, three further parameters were chosen in this work to strengthen the concept of likeness. The first input parameter was the dynamics of the neuritic outgrowth. In silico dynamics globally approximated the experimental one with a standard level of confidence (Welch t-test, p = 0.3504).

The second output parameter was the mean angle of alignment. Also in this case, statistical analysis showed the absence of significant differences (Welch t-test, p = 0.3571) between biological experiments and in silico simulations over the global time range (t = 6 ÷ 60 h from NGF administration), and for a standard level of confidence (95%).

Moreover, Figure 7E shows that most of the simulated neurites aligned to the main grating direction within 20° according to the biological data (respectively the 90% and 85%). Furthermore, in both biological experiments and in silico simulations, the value of 30° seemed to represent a cut-off value, over which the percentage of growing neurites was negligible (biological experiments) or null (in silico simulations).

The third output parameter was tortuosity, and quantitative comparisons were made with PC12 and primary cells [37]. In silico results were in good agreement not only with experimental values for both PC12 (difference ∼ 0.1%) and primary cells (difference ∼ 0.3%) [37], but also with the theoretical simulation of primary cells (difference ∼ 0.2%).

Therefore, the similarity between in silico and biological neurites was assessed using all these parameters: once the dynamics of neurites and the geometry of nanograting were inserted within in silico simulations, the angles of alignment and tortuosity were also in agreement with biological data.

This seemed to suggest that the global outputs (alignment, tortuosity) resulting from the coupling between dynamics and topography (inputs) were able to catch the main features of the real PC12 outgrowth on nanograting. As a consequence, this simple system, ruled by deterministic interactions and stochastic fluctuations, was able to approximate a much more complex cellular system.

In this study, both dynamics and angles of alignment were analysed along the whole interval of time, so the likeness was globally computed for all in silico simulations. This procedure was effective because the length of PC12 neurites had only small changes along the NGF phase (about 10 µm). Nevertheless, in the case of greater elongations in time and large variations in alignment, global likeness should be replaced by local likeness, and in silico values very close to experimental ones may be required for each experimental time point both for input dynamics and output alignments.

In conclusion, this work presented a synergistic procedure, which is a promising starting point towards more complex simulations accounting for also highly irregular dynamics and inhomogeneous outgrowth.

Thanks to its simplicity, this procedure can be used to implement inverse simulations coupled with different computational strategies (e.g., genetic algorithms [43]) and to approach a set of complex optimization problems, often with many possible solutions.

Indeed, just starting from a given set of optimal neuritic paths, many suitable surfaces respecting initial constraints and boundary conditions can be identified. Then, from this set, the best surface has to be found to optimize technological issues and time variant effects.

In addition, through inverse simulations, the specific behaviour of cells (PC12 or primary cells with a similar behaviour at the cell/surface length scale) can be inserted as “biological constraint” into design procedures together with other classic issues (e.g., surface technology, biocompatibility of materials).

This computational model could be also applied to dynamic problems related to the effectiveness of regenerative interfaces [17]: in this case, mechanical interferences between afferent (sensory), efferent (motor), and autonomic fibres largely decrease the reliability of the coupling between axons and neural interface. At first, indeed, many contacts are established between motor fibres and active borders of holes lying over the partition surface. Then, sensory fibres grow faster than motor fibres, causing compressive interferences within the holes. Since compressive stresses choke motor fibres, most of the remaining connections are with sensory fibres: this precludes access to the motor functions of patients.

A possible way to decrease these effects is to split motor and sensory fibres, conveying them to different partition surfaces (see Figure 1). To this aim, the dynamic characteristics of fibres (e.g., velocity of growth, degree of superposition, ability to branch, interaction forces between neurites) could be synergistically used together with static features (e.g., geometry and elasticity) within the presented framework. Finally, all these possibilities can be coupled with the action of chemical gradients [44], deriving from any set of sources in a chemical active environment, and making it possible to study the non linear superposition between chemical and topographic cues both in static and dynamic conditions.
